# Surgical Management of Advanced and Metastatic Renal Cell Carcinoma: A Multidisciplinary Approach

**DOI:** 10.3389/fonc.2017.00107

**Published:** 2017-05-31

**Authors:** Brian M. Shinder, Kevin Rhee, Douglas Farrell, Nicholas J. Farber, Mark N. Stein, Thomas L. Jang, Eric A. Singer

**Affiliations:** ^1^Section of Urologic Oncology, Rutgers Cancer Institute of New Jersey and Rutgers Robert Wood Johnson Medical School, New Brunswick, NJ, United States; ^2^Division of Medical Oncology, Rutgers Cancer Institute of New Jersey and Rutgers Robert Wood Johnson Medical School, New Brunswick, NJ, United States

**Keywords:** renal cell carcinoma, targeted therapy, cytoreductive nephrectomy, cytoreductive partial nephrectomy, lymphadenectomy, neoadjuvant, metastasectomy

## Abstract

The past decade has seen a rapid proliferation in the number and types of systemic therapies available for renal cell carcinoma. However, surgery remains an integral component of the therapeutic armamentarium for advanced and metastatic kidney cancer. Cytoreductive surgery followed by adjuvant cytokine-based immunotherapy (predominantly high-dose interleukin 2) has largely given way to systemic-targeted therapies. Metastasectomy also has a role in carefully selected patients. Additionally, neoadjuvant systemic therapy may increase the feasibility of resecting the primary tumor, which may be beneficial for patients with locally advanced or metastatic disease. Several prospective trials examining the role of adjuvant therapy are underway. Lastly, the first immune checkpoint inhibitor was approved for metastatic renal cell carcinoma (mRCC) in 2015, providing a new treatment mechanism and new opportunities for combining systemic therapy with surgery. This review discusses current and historical literature regarding the surgical management of patients with advanced and mRCC and explores approaches for optimizing patient selection.

## Introduction

Renal cell carcinoma (RCC) remains one of the most commonly diagnosed cancers in the United States, with 63,990 new cases and 14,400 deaths expected in 2017 (1). The majority of patients will present with organ-confined disease (2), and surgical resection of these tumors generally results in excellent long-term disease-free survival (DFS) (3). However, for patients with advanced or metastatic disease, survival rates are poor (4, 5). Approximately 20–30% of men and women with RCC present with metastatic disease, while 20–40% of those who undergo surgical resection for localized RCC will develop metastases, making this disease state a considerable challenge (6, 7).

Despite recent advances in systemic therapies, surgery remains an integral component in the treatment algorithm for aggressive disease phenotypes. In this review, we discuss the role of surgery in the overall management of locally advanced and metastatic renal cell carcinoma (mRCC).

## Cytoreductive Nephrectomy in the Cytokine Era

Cytoreductive nephrectomy (CN) is defined as the surgical removal of the primary renal tumor in the setting of mRCC. During the initial years of the cytokine era, controversy existed as to whether CN had any clinical benefit. Several early retrospective studies demonstrated an improved response to immunotherapy in patients undergoing debulking surgery compared with patients treated with cytokines alone (8, 9). Eventually, landmark Level 1 evidence supporting CN plus cytokine therapy with IFN-α in advanced RCC was established by the results of two prospective phase 3 randomized controlled trials: SWOG-8949 (Southwest Oncology Group) (10) and EORTC-3047 (European Organization for Research and Treatment of Cancer) (11). Both the SWOG and EORTC trials demonstrated a significant overall survival (OS) advantage and improved progression-free survival (PFS) in patients who underwent CN prior to cytokine therapy versus patients undergoing immunotherapy alone with IFN-α (10, 11). The SWOG trial evaluated 241 patients and showed a 3-month OS benefit in the nephrectomy group versus non-nephrectomy group (11.1 vs. 8.1 months, respectively) (10). A subsequent update of the SWOG data with 9-year follow-up again favored CN by showing a 3-month OS benefit in the nephrectomy group and a 26% reduction in death (12). Likewise, the EORTC study showed an even greater benefit in patients undergoing CN followed by IFN-α versus IFN-α alone. Time to progression [5 vs. 3 months, hazard ratio (HR) 0.60; 95% CI, 0.31–0.94; *p* = 0.04], and median duration of survival (17 vs. 7 months, HR 0.54; 95% CI, 0.36–0.97; *p* = 0.03) were significantly better in the patients who underwent CN (11). Importantly, CN was concluded to be safe, as both studies showed a very low perioperative mortality rate of less than 1%. A meta-analysis of the SWOG and EORTC data showed an OS of 13.6 months among patients who underwent CN plus IFN-α vs. 7.8 months for IFN-α alone, representing a 31% relative risk reduction in risk of death. Also, 95% of patients in combined analysis of the SWOG and EORTC data were able to receive IFN-α after surgery (median 19 days later) (13). Furthermore, an analysis of 5,372 people with mRCC from the Surveillance Epidemiology and End Results Program (SEER) database demonstrated a significant survival benefit of CN, with a 10-year OS of 12.7 versus 1.2% for those without surgery (14). As a result of these analyses, the role of CN was established as standard therapy for patients with mRCC in the cytokine era.

## CN in the Targeted Therapy Era

In the past decade, the systemic management of mRCC has changed significantly, as our understanding of the molecular biology of RCC has increased (15). Multiple-targeted therapies (TTs) that primarily inhibit VEGF (sunitinib, sorafenib, axitinib, bevacizumab, cabozantinib, lenvatanib) and mTOR pathways (temsirolimus, everolimus) have been approved for the management of mRCC (Table 1) (16). In light of the efficacy of TT agents, the utility of nephrectomy in patients with mRCC has been questioned. In fact, recent reports indicate declining utilization rates of CN in the TT era (17, 18). An analysis of the SEER database by Tsao et al. showed that the rate of CN performed was about 50% in patients with stage IV RCC until 2005, when TTs were approved by the FDA. There was then a steady decrease to 38% in 2008, possibly due to the yet unknown interaction between CN and TT and an unwillingness to subject patients to the morbidity of surgery given the benefits of TT (19). Currently, evidence supporting the role of CN in the TT era for the management of mRCC remains limited and is primarily based on retrospective series and administrative databases.

**Table 1:** **FDA-approved therapies for renal cell carcinoma with their pivotal trial parameters**.

Therapy	FDA approval	Treatment line	Mechanism of action	Route	Comparator arm	Primary endpoint
Interleukin-2 (20)	May 1992	First	Cytokine immunotherapy	IV	Phase II—none	ORR
Sorafenib (21)	December 2005	Cytokine failure	VEGFR, PDGFR, RET, KIT inhibitor	Oral	Placebo	OS
Sunitinib (22)	January 2006	First	VEGFR and PDGFR inhibitor	Oral	IFN-α	PFS
Temsirolimus (23)	May 2007	First	mTOR inhibitor	IV	IFN-α	OS
Everolimus (24)	March 2009	VEGFR failure	mTOR inhibitor	Oral	Placebo	PFS
Bevacizumab + IFN-α (25, 26)	July 2009	First	Anti-VEGF monoclonal antibody	IV + SC	IFN-α ± placebo	OS
Pazopanib (27)	October 2009	First or cytokine failure	VEGFR, PDGFR, KIT inhibitor	Oral	Placebo	PFS
Axitinib (28)	January 2012	Second	VEGFR inhibitor	Oral	Sorafenib	PFS
Nivolumab (29)	November 2015	Second	Anti-PD1 monoclonal antibody	IV	Everolimus	OS
Cabozantinib (30)	April 2016	Second	VEGFR, MET, AXL inhibitor	Oral	Everolimus	PFS
Lenvatinib + Everolimus (31)	May 2016	Second	VEGFR, FGFR, PDGFR, RET, KIT inhibitor + mTOR inhibitor	Oral	Everolimus or lenvatinib	PFS

*Modified with permission from Modi et al. (16)*.

Choueiri et al. (32) reported on 314 patients with mRCC treated with adjuvant anti-VEGF-targeted agents and concluded that those who previously underwent CN lived a median of 10 months longer (19.8 vs. 9.4 months, HR 0.44; 95% CI 0.32, 0.59; *p* < 0.01). Although patients who underwent CN had fewer negative predictors of survival (lower corrected serum calcium levels and a better Karnofsky performance status), when controlled for known prognostic factors, CN still demonstrated a significant OS benefit (HR 0.68, *p* = 0.04) (32).

Heng et al. used the International Metastatic Renal Cell Carcinoma Database Consortium (IMDC) to identify patients with synchronous mRCC treated with adjuvant-targeted therapy at the population-based level and examined survival in this group. Of 1,658 patients, 982 (59.2%) underwent CN. OS was significantly higher than in those undergoing surgery (20.6 vs. 9.5 months, *p* < 0.01), and, after adjusting for prognostic risk factors, patients undergoing nephrectomy had a 40% decreased risk of death (HR 0.60, 95% CI 0.52, 0.69; *p* < 0.0001) (33).

Similarly, Hanna et al. (34) recently reported on 15,390 patients diagnosed with primary mRCC from the National Cancer Database (NCDB) treated with at least one TT, 5,374 (35%) of whom also underwent CN. The median time to death was significantly longer in the CN group versus those who did not undergo surgery (17.1 vs. 7.7 months, respectively; *p* < 0.001), and the 3-year OS rates were 62.7% for those who underwent CN and just 9.8% in the non-CN group. Additionally, after adjusting for various covariates such as age, Charlson Comorbidity Index (CCI), T stage, and other factors, multivariate Cox regression analysis revealed CN patients had a lower risk of death (HR 0.49, *p* < 0.001). Finally, a recent meta-analysis that included 39,953 patients across 12 studies found a reduced risk of death (HR, 0.46; CI, 0.32–0.64; *p* < 0.01) for patients who were treated with CN and TT compared to just TT alone (35).

Despite this evidence, careful patient selection remains critical. Mathieu and colleagues found in their cohort of 351 patients with mRCC that the benefit of CN in combination with adjuvant TT was lost when examining patients with poor performance status (36). Among the entire cohort, median OS was significantly greater for those who underwent CN and TT versus TT alone (38.1 vs. 16.4 months, *p* < 0.001, respectively). For patients with an ECOG score of 2 or 3, however, this significance was lost. Furthermore, there was no difference in survival for patients with poor MSKCC risk scores. This is congruent with the subgroup analysis in the study by Choueiri et al. described above, where patients with a poor Karnofsky performance status (<80%) did not have an improved OS (*p* = 0.08). These results suggest that while CN may be favorable in the mRCC setting, patients who have other significant comorbid conditions, harbor brain or bone metastases, have a high burden of disease outside of the kidney, or a poor performance status might be better served by immediate systemic therapy instead of CN. These findings also illustrate the importance of proper CN patient selection.

## CN Patient Selection

Cytoreductive nephrectomy can be a challenging operation with significant potential morbidity, especially in older patients, and has been associated with increased complication and perioperative mortality rates (37, 38). Using the SEER database, Cloutier et al. (39) demonstrated a 30-day mortality of 4.2% in mRCC patients (all T stages) compared with just 0.3 and 1.3% for patients with T_1-2_N_0_M_0_ and T_3-4_N_0-2_M_0_ disease, respectively, for those who underwent partial or radical nephrectomy. However, this rate reached 10.5% in the subset of patients aged 80 years or older. Similarly, Sun et al. (40) confirmed that elderly patients (>75 years) were 2.2-fold more likely to experience perioperative mortality than younger patients (<75 years) (4.8 vs. 1.9%, *p* < 0.001). Thus, accurately identifying patients who stand to benefit from the operation is imperative.

Early retrospective studies on CN reported an overall mortality rate of up to 2.5%, with up to 40% of patients being unable to subsequently receive systemic cytokine therapy for a variety of reasons, mainly perioperative complications, perioperative death, or tumor progression during the postoperative period (41, 42). Likewise, Bennett and colleagues evaluated 30 patients with stage IV RCC and found 23 (77%) were unable to receive systemic cytokine therapy after CN due to either perioperative death, cardiovascular insufficiency, rapid disease progression during the postoperative period, prolonged surgical morbidities, compromised renal function, or mental distress (43). The results of the original SWOG and EORTC trials themselves demonstrated that up to 25% of patients in both groups progressed rapidly and died within 4 months, suggesting that a significant proportion of patients may not benefit from surgery (10, 11). It is clear that the morbidity associated with CN should not be considered negligible, and great effort should be made to define the optimal profile of patients for surgery.

As the oncologic course of patients with mRCC can vary considerably, several predictive models for patients who may benefit from CN have been developed. Fallick and colleagues (41) developed several criteria for patient selection for CN: (1) tumor debulking representing greater than 75% of the total tumor burden; (2) no central nervous system, bone, or liver metastases; (3) predominantly clear cell histology; (4) adequate pulmonary and cardiac function; and (5) ECOG performance status of 0 or 1. However, other reports have demonstrated that the presence of bone metastases is not a relative contraindication to CN unless the patient has a significant burden of disease and that patients with non-clear cell histology may still benefit from CN (44). Furthermore, these studies investigated patients who received IFN-α, making its utility in the current TT era unknown.

To evaluate factors influencing survival among patients undergoing CN in the TT era, Culp et al. (45) retrospectively analyzed 566 patients who underwent CN in comparison to 110 patients receiving targeted therapy alone. Multiple clinical parameters were compared between those surviving <8.5 months (which was defined as no benefit from CN), and those surviving longer. The authors identified 7 seven variables that were significantly associated with a shorter survival: serum albumin below lower limit of normal (LLN), LDH above upper limit of normal (ULN), a clinical tumor classification of T3 or T4, symptoms from a metastatic site, presence of liver metastasis, presence of retroperitoneal adenopathy, and presence of supradiaphragmatic adenopathy. The authors concluded that patients with 3 three or less of these negative predictors might benefit from CN compared to systemic TT alone (HR, 0.39; 95% CI, 0.31–0.48; *p* < 0.001), while those with 4 or more would not benefit from CN (HR, 0.89; 95% CI, 0.64–1.24; *p* = 0.499). Although these results are limited by the study’s retrospective nature with no external validation, this was the first large-scale analysis of multiple preoperative clinical parameters that may potentially predict outcomes following CN. Following this, Margulis et al. (46) developed a multivariable model to predict cancer cancer-specific survival at 6 and 12 months following CN (46). The preoperative nomogram assigns a point score from 0 to 100 based on a patient’s patient’s albumin and LDH. This score is then used to predict the 6 and 12- month risk of death from kidney cancer. Though the model demonstrated good calibration and a discrimination of 0.76 when applied to an internal validation set, further external validation is still required.

In another attempt to identify risk factors that can be used to optimize patient selection for CN, Heng et al. described the use of the IMDC criteria for selecting patients who would benefit from CN. These criteria are the six preoperative risk factors of Karnofsky performance score <80, hemoglobin < LLN, neutrophils > ULN, platelets > ULN, diagnosis to targeted therapy <1 year, and serum corrected calcium > ULN. Patients who had four, five, and six of these risk factors did not derive any significant benefit from CN in an analysis of the IMDC database of patients who were treated with CN and a TT. Additionally, the interaction between the number of IMDC factors patients had and nephrectomy status was statistically significant *p* = 0.0005, supporting their use in patient selection (33).

Abel et al. (47) demonstrated the importance of time to maximal response to TT as a measure of predicting eventual oncologic outcomes. Achieving a decrease in primary tumor size of ≥10% was an independent predictor of OS (HR, 0.43, *p* = 0.007), while a decrease within the first 60 days of treatment was an even stronger predictor of survival on multivariate analysis (HR, 0.26; *p* = 0.031). These findings have led many to advocate the use of the initial response to systemic therapy as a “litmus test” to identify patients who could benefit from CN (48).

Pathology-derived predictors have also been evaluated for associations with post-CN survival. Sarcomatoid and non-clear cell histology have consistently been associated with worse survival outcomes (44, 49). In one report of 417 patients undergoing CN, 62 patients with sarcomatoid features had a median survival of 4.9 versus 17.7 months for those without these features (*p* < 0.001) (50). Culp et al. (45) also found a HR for survival of 1.4 (95% CI, 1.08–1.80; *p* = 0.011) for the presence of sarcomatoid features in their analysis of factors affecting survival after CN. However, this same group later demonstrated that percutaneous biopsy of a primary renal mass was only able to identify sarcomatoid features in 12% of tumors later found to have such features after nephrectomy, suggesting it has only marginal utility to guide preoperative clinical decision making (51).

Patients with non-clear cell mRCC have frequently been shown to have worse survival outcomes after CN compared to those with clear cell histology. Kassouf et al. (44) reported significantly worse survival for 92 patients with non-clear cell mRCC compared to 514 patients with clear cell histology: 9.7 versus 20.3 months, respectively (*p* = 0.003). Patients with non-clear cell mRCC had a higher rate of sarcomatoid features (23 vs. 13%, *p* = 0.026), but even patients with non-clear cell mRCC without sarcomatoid features had shorter survival of 14 months compared to 23.1 months for patients with clear cell RCC with sarcomatoid features (*p* = 0.017). It is unclear whether this is because of non-clear cell mRCC’s lack of response to cytokine therapy or more aggressive biology (52). Non-clear cell mRCC also has shown a poorer response to TT compared to clear cell mRCC (53).

The tumor burden of RCC has previously been shown to be predictive of overall and PFS (54). In light of this, the percentage of tumor burden removed by nephrectomy, classically termed fractional percentage of tumor volume (FPTV), may provide a relatively easy way to estimate the survival benefit of CN. Recent reports suggest a threshold of >90% FPTV to achieve improved PFS and OS. For instance, Pierorazio et al. (55) described 55 patients undergoing CN with or without metastasectomy, of which 45 had >90% FPTV removed. The patients with a FPTV removal of >90% had improved DSS (HR 0.29, *p* = 0.02) on multivariate analysis when controlling for volume of metastatic disease and multifocal lesions. Similarly, Barbestefano et al. (56) demonstrated a shorter PFS for patients with FPTV removal of <90% in a series of 46 patients with clear cell RCC followed by targeted therapy. No patients in this study were reported to have undergone metastasectomy. The 15% of patients with FPTV removal of <90% had a higher risk of disease progression (HR 4.8, *p* < 0.001) on multivariate analysis that controlled for known predictive variables.

## Cytoreductive Partial Nephrectomy

Partial nephrectomy, or nephron-sparing surgery (NSS), is considered the treatment of choice for localized small renal masses, with oncological outcomes comparable to RN in the management of RCC (57). As preoperative renal dysfunction is present in a substantial number of patients with RCC, and radical nephrectomy has been shown to increase the risk for chronic kidney disease (CKD), surgical approaches that maximize postoperative renal function should be considered (58). There is considerable evidence in the literature that the tumor characteristics, rather than surgical approach, determine CSS and OS (59, 60). For example, Margulis et al. (61) compared the oncological efficacy of partial nephrectomy versus radical nephrectomy in patients with locally advanced pT_2_-T_3b_N_0_M_0_ RCC. In this comparison of 34 patients undergoing partial nephrectomy versus 567 patients undergoing radical nephrectomy, CSS was similar between the two surgical approaches (78 vs. 74%, respectively, *p* = 0.113), though the 5-year recurrence free survival was improved (82 vs. 62%, *p* < 0.012). Moreover, after adjusting for tumor stage, grade, and histology, there were no differences in outcomes.

Although CN is now considered an integral component of the mRCC treatment algorithm, the role of cytoreductive partial nephrectomy (CPN) in this same disease context is not well established, and the current body of literature supporting its use is limited. In 1996, Krishnamurthi et al. (62) retrospectively reviewed 15 patients who underwent CPN for mRCC due to a solitary kidney or CKD. Overall renal function was preserved in 14 out of the 15 patients, as only 1 went on to require hemodialysis. Although this study was limited by its inability to calculate pooled CSS for all the subjects due to the heterogeneity of patient characteristics, burden of metastasis, and adjuvant treatment received, these results nonetheless suggested a potential utility for CPN. Krambeck et al. (63) later reviewed the Mayo Clinic Nephrectomy Registry between 1970 and 2002. Sixteen patients who underwent CPN for mRCC with a median follow-up period of 18 months were compared with 404 patients who underwent RN for mRCC. This study demonstrated that survival of patients undergoing CPN was significantly better than those undergoing RN, with 1-, 3-, and 5-year CSS for the CPN group of 81.3, 49.2, and 49.2%, respectively, compared to 50.5, 21.1, and 12.8%, respectively, for the RN group (*p* = 0.013). One major limitation of this study was that 87.5% of the patients in the CPN group underwent complete resection of all metastatic sites compared with only 22.5% from the RN group, which likely biased the final results. In a multi-institutional study, Hutterer et al. (64) reviewed the nephrectomy database of 17 institutions between 1984 and 2001. Of the 777 mRCC patients treated with nephrectomy, 5.8% had undergone CPN with a mean follow-up period of 1.8 years. In both a matched and unmatched analysis, a higher, though statistically insignificant, rate of RCC-specific mortality was seen in those who underwent RN.

Capitanio et al. (65) examined the effect of CPN on DSS in comparison to RN in a population-based study using the SEER registry. From 1988 to 2004, a total of 2,043 patients with metastatic disease underwent CN, with a median follow-up period of 23.5 months. CPN was performed in 2.2% of the patients and each of the 46 CPN patients were matched with up to four RN patients for tumor size and race. In this matched population, the 1-, 2-, 5-, and 10-year CSS estimates were 58.8, 44.8, 27.1, and 19.7%, respectively, for RN patients versus 79.2, 60.9, 40.2, and 40.2% for CPN patients, respectively. This represented a marginally significant association between CPN and CSS (HR 1.78, *p* = 0.05). Likewise, Hellenthal et al. (66) identified 56,011 RCC patients in the SEER database between 1988 and 2005. Fifteen percent of the patients had metastatic disease at presentation. Of these patients with mRCC, 35% underwent surgical treatment with only 2.4% having CPN. On multivariate analysis, it was found that patients undergoing CPN were 0.5 times less likely to die of any cause and 0.48 times likely to die of RCC than those who underwent RN (95% CI 0.27–0.91, *p* < 0.024 and 95% CI 0.25–0.94, *p* < 0.031, respectively). Notably, the authors found that tumor size was significantly associated with both overall and cancer-specific survival. This may be partly responsible for the survival benefit seen with CPN, as only 1% of patients who had a primary tumor size ≥7 cm underwent this surgery.

Even in the absence of high-quality data supporting the use of CPN for patients with mRCC, it could be considered for tumors amenable to such an approach. Established prognostic factors should also be used to assist with patient selection. Additionally, patients for whom CN is not feasible due to preexisting renal impairment or a solitary kidney can be expected to derive benefit from a nephron-sparing approach, especially in cases in which the primary tumor is small.

## Ongoing Randomized Clinical Trials Addressing CN

To prospectively examine the role of CN in the context of adjuvant-targeted therapy, two large randomized trials are currently ongoing. The Clinical Trial to Assess the Importance of Nephrectomy (CARMENA; NCT00930033) examines nephrectomy followed by sunitinib treatment compared with sunitinib alone in patients with mRCC. This phase III non-inferiority study was initiated in 2009 and enrolls patients with clear cell mRCC and an ECOG performance status of 0 or 1. The trial has an estimated study completion time of February 2018. Additionally, the EORTC Immediate Surgery or Surgery after Sunitinib Malate in Treating Patients with Metastatic Kidney Cancer (SURTIME; NCT01099423) trial assesses the timing of nephrectomy relative to treatment with sunitinib in patients with resectable mRCC. SURTIME was initiated in 2010 and randomizes patients with mRCC to either a nephrectomy followed by sunitinib or to three courses of sunitinib therapy with subsequent nephrectomy. The primary and secondary endpoints of the SURTIME trial are PFS and OS, respectively.

## Neoadjuvant Systemic Therapy for mRCC

Neoadjuvant therapy is frequently used in the treatment of various malignancies to reduce the primary tumor mass and improve the outcome of definitive surgical treatment. In the era of cytokine-based systemic therapy for RCC, there was little role for the neoadjuvant approach. Cytokines such as interleukin-2 and IFN-α were shown to confer an overall increase in survival for patients with metastatic disease, but their benefit in conjunction with surgery or their use in reducing the size of the primary tumor was rarely reported (67). The expansion in the number of TTs and their effects on tumor biology have renewed interest in neoadjuvant treatments for mRCC. Potential benefits of this include improving surgical resectability of the kidney for locally advanced tumors as well as facilitating the ease to which CPN can be performed. Additionally, a lack of response to neoadjuvant therapy may help identify individuals with aggressive disease that would not benefit from nephrectomy, thus avoiding surgical morbidity with little benefit (68).

Powles et al. (69) recently published a report on the use of pazopanib in the neoadjuvant setting for patients with mRCC. A single arm phase II study was conducted that included 104 treatment-naïve patients, although four of these patients could not be analyzed after they dropped out due to treatment related toxicities. Pazopanib was given for 12–14 weeks prior to CN and then continued afterward until disease progression. Clinical benefit, or no evidence of disease progression, was seen in 84% of the patients, though only 63% of patients underwent surgery. The most common reasons for not undergoing surgery included disease progression (*n* = 13), patient choice (*n* = 9), and inadequate fitness for surgery (*n* = 5). Overall, the median PFS and OS was 7.1 months (95% CI, 6.0–9.2) and 22.7 months (95% CI, 14.3-not estimable), respectively (69).

Thomas et al. (70) prospectively evaluated 19 patients with advanced RCC who were deemed unsuitable for surgical intervention due to either locally advanced disease or extensive metastatic burden. Patients received sunitinib (50 mg; 4 weeks on and 2 weeks off) until the primary tumor was determined to be operable, the disease progressed, or there was unacceptable toxicity. No patients experienced a complete response after a median follow-up of 6 months with the median number of cycles of sunitinib as 2. However, 8 patients (42%) were found to have average primary tumor shrinkage of 24% (range 2–46%). At the time of follow-up, 4 patients (21%) had undergone nephrectomy, although 5 other patients had died due to disease progression. Interestingly, one of the patients who underwent surgery did not have any reduction in primary tumor size, but rather demonstrated a considerable response in metastatic disease burden, thereby making cytoreductive surgery a reasonable treatment option.

A similar study by Rini et al. (71) investigated 30 patients with unresectable RCC, 19 of which had distant metastases. After receiving a median of three cycles of sunitinib, there was a median decrease of 22% in the primary tumor size and 13 patients (45%) were able to undergo surgical resection. Notably, 9 patients were able to have a partial nephrectomy. Only 3 of the patients who were able to have surgery had metastatic disease, suggesting that there may be significant heterogeneity between the primary tumor and metastatic sites. Several other studies have reported on the feasibility of primary tumor downsizing with various neoadjuvant TT, but no single drug appears to be markedly more efficacious than the others. The range of median tumor diameter reduction reported by these studies was 9.5–28.3% (Table 2) (70–81). These results highlight the important role that neoadjuvant treatment can play in advanced and metastatic RCC and suggest that it should be considered for carefully selected patients with unresectable tumors.

**Table 2:** **Results of selected clinical trials evaluating effect of neoadjuvant TT agents on primary tumor size**.

Reference	Participants	Drug	Percent metastatic disease	Median tumor shrinkage (%)
van der Veldt et al. (72)	17	Sunitinib	100	12
Thomas et al. (70)	19	Sunitinib	79	24
Cowey et al. (73)	30	Sorafenib	43.3	9.6
Hellenthal et al. (74)	20	Sunitinib	20	11.8 (mean)
Silberstein et al. (75)	12	Sunitinib	42	21 (mean)
Powles et al. (77)	66	Sunitinib	100	13
Rini et al. (71)	28	Sunitinib	66	22
Karam et al. (78)	23	Axitinib	0	28.3
Rini et al. (80)	25	Pazopanib	0	26
Lane et al. (79)	72	Sunitinib	40	18
Zhang et al. (81)	18	Sorafenib	39	20.5 (mean)
Powles et al. (69)	100	Pazopanib	100	14.4

Primary tumor reduction is also of interest to preserve renal function by enabling partial nephrectomy to be performed instead of radical nephrectomy. In order to estimate the volume of kidney that could be preserved by neoadjuvant pazopanib, Rini et al. (80) found a decrease in the R.E.N.A.L. score in 71% of tumors from 25 patients with locally advanced RCC. This allowed for 6 of 13 NSS ineligible patients to then undergo NSS. Similarly, it was found that more patients became eligible for partial nephrectomy after axitinib treatment, and 14 of 24 patients had an increase in the amount of observers who determined NSS was feasible (84). It should be noted that the ability to perform a partial nephrectomy is highly subjective and can differ between surgeons, suggesting the utility of neoadjuvant treatment in facilitating NSS be evaluated on an individual basis. Future studies are needed with stricter criteria for NSS feasibility in order to definitively assess the ability of neoadjuvant therapy to convert RN to NSS.

## Perioperative Safety of Neoadjuvant-Targeted Therapy

Historically, there have been concerns of the risks that neoadjuvant treatment with TTs pose in the perioperative setting. However, to date, surgery after neoadjuvant TT has been performed safely with no difference in overall surgical mortality and morbidity (69, 82, 85). One area of concern has been reports of delayed postoperative wound healing, though conflicting data currently exists. Some studies have shown wound healing rates after neoadjuvant therapy consistent with historical controls of upfront nephrectomy (86, 87), while others had concerns with delayed wound healing in up to 24% of patients (69, 83, 85, 88). To prevent wound complications, investigations of when the last dose of targeted therapy should be given before nephrectomy have been done. Thomas and colleagues suggested that withholding the drug for 2–3 half lives before and after surgery could serve to minimize these adverse effects (82). Still, no consensus guidelines on the optimal time to stop neoadjuvant therapy prior to surgery currently exist. Regardless of when it is stopped, careful surgical technique with attention to wound closure should be a priority.

Another concern of neoadjuvant-targeted therapy has been the phenomenon of tumor “rebound.” While TKIs and VEGF-A decrease the vascularity of tumors, cessation of treatment can lead to rapid intratumoral angiogenesis and thus progression of disease. The mechanism of these changes has not been fully elucidated. Initial preclinical studies of such drugs suggested that an upregulation of tumorigenic and proinflammatory cytokines and growth factors, along with changes to the tumor microenvironment that increase the metastatic potential, may be responsible (89). However, few clinical studies have been done to investigate this, and much of the early evidence was from case reports (90, 91). One study looked at primary tumor tissues of patients treated with sunitinib or bevacizumab and compared them with those who were untreated. While the investigation found that sunitinib-treated tumors showed suppression of angiogenesis by analysis of several angiogenic markers, it also found an increased number of growing endothelial cells, suggesting that cessation of VEGF TKIs could cause quick revascularization of tumor after repression (92). Powles et al. (93) pooled data from three phase II clinical trials in which sunitinib (two studies) or pazopanib (one study) was given prior to nephrectomy in patients with mRCC. In each of the studies, a treatment break of 4–5 weeks was planned in order to minimize the risk of wound healing complications. Of the 62 patients who underwent the procedure, 23 (37%) had radiographic evidence of progression, 77% of which had new metastatic sites, at a median follow-up of 6.3 weeks after the procedure. The study found that 70% of the patients who progressed during structured treatment break could be stabilized by reimplementation of targeted therapy. Nonetheless, the patients who progressed had a significantly shorter OS than those who did not (HR: 5.56, *p* < 0.01). The authors were unable to find any factors that could be used to predict those that would progress during treatment break, although those who did progress tended to have tumors with a higher Fuhrman grade (93).

In summation, studies looking at the safety of surgical resection of kidneys in RCC after the use of various TTs seem to agree that surgery after therapy is relatively safe with tolerable morbidity and mortality outcome. Though neoadjuvant therapy may increase the risk for delays in superficial wound healing and withdrawal of therapy may result in tumor proliferation, these concerns should be weighed against the clinical benefit derived from such therapeutic strategies.

## Neoadjuvant Systemic Therapy for Caval Thrombus Due to Advanced RCC

Tumor thrombus extension into the IVC is a potential consequence of advanced RCC. Surgery, while often technically challenging, has historically been shown to improve long-term survival in patients without metastases (94, 95). Data have also suggested that the level of tumor thrombus correlates with higher perioperative complication rates (96, 97). Neoadjuvant therapy could potentially allow for a less complicated surgical procedure by downgrading the tumor thrombus level (98). While individual case reports have indicated some success, the overall benefit of neoadjuvant therapy for the downsizing of tumor thrombus has not yet been established. A more recent study looked at neoadjuvant sorafenib for treatment of high-risk RCC and found that four of the five patients with tumor thrombus were able to downgrade thrombus level (81). However, one of the few case series to look at neoadjuvant-targeted therapy for the downsizing of caval thrombus found that only 1 of 24 patients was able to have a significant enough downsizing to change the surgical procedure (99). This report found only 44% of patients had a decrease in tumor thrombus height, with a decrease of 1.5 cm. Similarly, another small case series of 14 patients saw only 1 patient decrease in thrombus level with another patient increasing in thrombus level (100).

Although no conclusions can be drawn from this very limited data, physicians might consider such a therapeutic option in select cases, especially when the patient’s performance status or comorbidities make surgery especially high risk. Additionally, the inherently complex nature of caval thrombus tumor removal highlights the need for a multidisciplinary team approach. Depending on the precise location of the tumor and extent of the thrombus, collaboration with vascular or thoracic surgery colleagues and cardiac anesthesiology should be considered in order to ensure the greatest likelihood of success.

## Lymphadenectomy for Advanced RCC

Lymph node involvement in RCC is historically associated with a poor prognosis (101, 102). Even in the setting of metastatic disease, nodal metastases have been associated with worse outcomes (103, 104). While lymph node dissection (LND) can provide diagnostic information, the low incidence of lymph node metastases in the absence of clinical suspicion, the lack of a consistent definition and template of LND, and the lack of strong evidence supporting a survival advantage for LND, have called into question the appropriate use of LND (105–107).

Nearly five decades ago, Robson and colleagues suggested the use of LND in conjunction with radical nephrectomy as a possible reason for their higher reported OS in patients with RCC (108). Since then, various studies have looked at the efficacy of LND as a treatment option and several have reported a possible benefit (102, 103, 109–111). One retrospective study on node-positive patients, who did (*n* = 112) and did not (*n* = 17) undergo LND as part of their radical nephrectomy, reported a statistically significant survival advantage on univariate analysis (*p* = 0.0002) with a median survival benefit of about 5 months for those who underwent LND (103). Moreover, Whitson and colleagues found a positive correlation between the number of nodes removed and cancer-specific survival in node-positive patients (111). Further analysis of this same dataset by Sun and colleagues concluded that the extent of LND did not correlate with CSS for pN+ patients (112). A more recent study reported a survival benefit associated with nodal yield (102). Each additional lymph node removed resulted in a 3–19% increase in CSS for patients with tumors larger than 10 cm, pT3c and pT4 tumors, or evidence of sarcomatoid features (102). Even if LND was therapeutically beneficial to pN+, the incidence of pN+ without clinical suspicion ranges 3.3–4.1%, which would make the number of patients needed to treat for a potential benefit high (105, 113, 114).

Conversely, several retrospective studies have shown there to be no survival benefit for LND in RCC (103, 112, 115, 116). Additionally, an EORTC phase III randomized control trial found no significant difference in OS, time to disease progression, or PFS between those who received a radical nephrectomy plus complete LND versus those who received radical nephrectomy alone (105). Although the RCT did not find a therapeutic benefit in LND, the study only examined patients with surgically resectable tumors (majority were pT2) who were found to be N0M0 on preoperative imaging and reported N+ disease in only 4% of subjects who underwent LND. While clinical node status detects a majority of pathological node-positive cases, it is well understood that regional lymphadenopathy is frequently due to inflammatory changes and not RCC metastases (117). For the improvement in identification of RCC patients at high risk for lymph node metastases, several indicators have been created (Table 3) (114, 118–121).

**Table 3:** **Selected tool used in the identification of renal cell carcinoma patients at high risk for lymph node metastases**.

Reference	Predicted accuracy (AUC) (%)	Factors
Blute et al. (118)	N/A	Nuclear grade 3/4, sarcomatoid component, tumor >10 cm, tumor pT3/pT4, histological tumor necrosis

Hutterer et al. (119)	78.4	Age, symptoms (systematic, local, asymptomatic), tumor size

Capitanio et al. (114)	86.9	Clinical T3-T4, clinical node status, presence of metastases, clinical tumor size

Babaian et al. (120)	89.0	ECOG performance score, clinical node status, local symptoms, lactate dehydrogenase

Gershman et al. (121)	85.0	LN short axis, radiographic perinephric/sinus fat invasion

This discrepancy in the literature may be attributed to the variation and lack of standardization on the extent and location of dissection. Typically, the right renal lymphatics drain into paracaval, precaval, interaortocaval, and retrocaval lymph nodes, while the left renal lymphatics drain into preaortic, paraaortic, and retroaortic lymph nodes (123). Although a limited LND of the hilar nodes would be the most convenient for removal and sampling, nodal metastases outside of this region are frequently seen without the presence of hilar metastases (106, 107). Specific dissection templates have been described in the literature (106, 108, 124–126). However, there has been no consensus on the most favorable approach. Regardless, optimizing the amount of lymph nodes examined should be paramount. Terrone and colleagues found that increasing nodal yield also increased the percentage of positive nodes found and concluded that more than 12 nodes were needed to be examined for sufficient staging (127). Likewise, a more recent study by Capitanio et al. (122) calculated the number of nodes required to reach a 90% chance of finding a positive node for each risk group previously established by the Mayo clinic (118). For low-, intermediate-, and high-risk groups, it was calculated that 13, 16, and 21 nodes, respectively, were needed to reach the 90% threshold (122).

Currently, the usage of LND with nephrectomy has been decreasing (128), which might be due in part to refinements in cross-sectional imaging that have improved our ability to clinically stage patients at risk of nodal disease. With mixed results in retrospective studies of LND as a therapeutic measure, and the only RCT showing no survival benefit, more prospective studies are needed to evaluate the efficacy of LND and stratify patients by who may and may not benefit from it. Furthermore, standardization of the LND template will be needed to create consistency in both clinical practice and future trials.

## Metastasectomy for RCC

As previously described, the introduction of TTs into treatment pathways has significantly improved OS in the setting of mRCC. Despite the benefits offered by systemic therapies, surgical resection of distant metastatic lesions, or metastasectomy, remains an option for carefully selected patients. Though the effect of such treatment has been highlighted in several retrospective analyses, there is a lack of level 1 evidence owing to the absence of randomized clinical trials.

Several early studies found resection was often feasible and effective in patients presenting with solitary metastasis (129, 130). The role of metastasectomy was later expanded to include patients with metastatic lesions in multiple organs. A retrospective analysis, performed prior to the TT era, of 278 patients with mRCC found the 5-year OS rate to be 44% for patients undergoing curative resection of metastatic tumors (131). Predictably, a higher disease burden was associated with poorer outcomes, as the 5-year OS rate was significantly lower in those with multiple sites of metastasis compared to those with a single site (29 vs. 54%, *p* < 0.001). Of note, for patients who did not undergo any surgical resection, the 5-year OS rate was only 11%, without any systemic therapy.

Alt et al. (132) was able to demonstrate a significant survival benefit for complete metastatic resection when the number of metastatic lesions was either 2 or ≥3 (*p* < 0.001 for both). Overall, patients who did not have any resection of metastases in this study had a 3-fold increased risk of death from RCC, even after controlling for the number, location, and timing of metastases, as well as the patients’ ECOG status (132). Regardless of disease burden, the value of complete metastasectomy compared to incomplete resection has been established. In the previous analysis, the 5-year estimated CSS rate was significantly higher for those who had complete versus incomplete resection of metastases (49.4 vs. 23.7%, respectively), though both were significantly higher than in patients who had no resection at all (8.9%) (132). Furthermore, Naito et al. (133) evaluated 556 patients with mRCC who had either complete or incomplete resection of their metastatic sites and showed a survival benefit of more than 70 months for patients who underwent complete resection versus incomplete (109.8 vs. 31.9 months; *p* < 0.001). This is consistent with work from Daliani and colleagues (134), who reported a median survival time of 5.6 years after complete resection versus 1.4 years for incomplete resection. A meta-analysis found a median 40.8-month longer OS and cancer-specific survival benefit of 14.8 months for complete resection (135). However, the existing data used for this analysis may reflect a selection bias of healthy patients for complete metastasectomy and the utilization of incomplete resection for palliative, not curative, effects. Addressing this issue, Zaid et al. (136) recently performed a similar meta-analysis of studies to compare complete versus incomplete metastasectomy or no surgery at all. They found comparable HRs for overall mortality in patients who had a complete metastasectomy, regardless of whether the study did or did not adjust for baseline performance status (2.59 vs. 2.16, *p* = 0.27, respectively). Overall, the pooled adjusted HR of 2.37 (95% CI, 2.03–2.87; *p* < 0.001) suggests that complete metastasectomy is advantageous when clinically feasible.

Combination treatments with systemic and surgical modalities have been investigated in order to enhance outcomes in this population. In the cytokine therapy era, several small retrospective series showed this was a reasonable approach (137–140). Later, Karam et al. (141) retrospectively analyzed 22 patients treated with targeted therapy and metastasectomy. They included those who had at least one cycle of targeted therapy prior to complete metastasectomy. At a median follow-up of 109 weeks after surgery, all but one of the patients was alive and at a median of 43 weeks and 50% had no tumor recurrence. Importantly, they noted only four patients had a total of six perioperative complications (four chylous ascites, one atrial fibrillation, and one ileus), which all were corrected with appropriate therapy. This interaction between metastasectomy and adjuvant systemic therapy must be examined further in well-designed clinical trials. One such trial, EGOG 2810 (NCT01575548) is a phase III study that is randomizing patients who have no evidence of disease following metastasectomy to receive either pazopanib or placebo using a primary outcome of DFS.

Renal cell carcinoma most often metastasizes to the lung, bone, lymph nodes, liver, pancreas, adrenal, and brain, with lungs the most common site (142). The 5-year OS rates for patients after pulmonary metastasectomy have been reported to range from 36 to 54%, with solitary metastatic lesions a good prognostic indicator for survival (143). Metastases to the pancreas have also been reported to have favorable outcomes following metastasectomy, with 5-year survival rates as high as 88% (144). In fact, a recent report found no statistically significant difference in recurrence free survival after complete metastasectomy in patients with either adrenal, liver, lung or pancreatic metastases (145). On the other hand, brain metastases have been reported in 2–17% of patients with mRCC (146–149). Outcomes are poor in these patients, with median OS of 10.7 months and 5-year survival rates of only 12% (150). Treatment for these patients is often palliative, and surgery, radiotherapy, stereotactic radiosurgery (STRS), and systemic treatments are frequently used in combination. Nieder and colleagues analyzed patients with metastatic RCC to the brain, and found median OS was greater in the subset of patients that underwent surgery with or without STRS versus those who had whole-brain radiotherapy alone (10.1 vs. 4.1 months) (151). Conversely, Ikushima et al. (152) compared metastasectomy plus radiotherapy with STRS, or conventional radiotherapy alone. Patients that underwent STRS had the highest median survival of 25.6 months, while the metastasectomy and conventional radiotherapy groups were 18.7 and 4.3 months, respectively. While this study suggests STRS should initially be considered for these patients, overall the data are quite limited and reinforce the need for a multimodal approach.

As mentioned previously, sarcomatoid differentiation accounts for 1–8% of renal tumors and has been shown to confer a poor prognosis for patients with RCC. Unfortunately, therapeutic options are limited for these patients as treatment with conventional chemotherapy, cytokine-based immunotherapy, or TTs do not provide much benefit (153–155). To identify whether metastasectomy offered a survival advantage to patients with sarcomatoid mRCC, Thomas et al. (156) identified 273 patients from an institutional database who underwent nephrectomy and were found to have sarcomatoid RCC before developing metastases. These patients then underwent metastasectomy of resectable sites. A control group of patients who did not receive metastasectomy were matched to these patients based on ECOG performance status, age, histology, pathological stage, and nodal status. For patients with synchronous metastases, median OS was 8.4 months for those who underwent metastasectomy and 8.0 months in the non-metastasectomy group (*p* = 0.35). Similarly, an observable, albeit insignificant, difference was seen in those with asynchronous metastases. The median OS in this asynchronous metastasectomy group was 36.2 months, compared to 13.7 months in the non-metastasectomy group (*p* = 0.29) (156). Even though this study did not find a significant survival benefit for metastasectomy in patients with sarcomatoid mRCC, it might still be considered in carefully selected patients, such as for palliative purposes.

## Future Directions

Locally advanced and metastatic RCC continues to present a challenge for providers as optimal predictive models and treatment pathways for this patient population have yet to be defined. Refinements in functional imaging, genomic sequencing of the primary tumor/metastasis, and biomarker/nomogram development offer opportunities to close some of the knowledge gaps in this disease space (157–159). Additionally, elucidating the role of immune checkpoint inhibitors in this patient population is critical. Therefore, clinical trial participation should be considered for each patient. For example, several recently opened high-priority trials using checkpoint inhibitors are briefly described below.

PROSPER is a phase III trial designed to evaluate the effects of perioperative nivolumab on recurrence-free survival (NCT03055013). Patients with clinical stage ≥ T2NxM0 or TanyN+ with tumors amenable to either radical or partial nephrectomy will be randomized to receive either surgery with neoadjuvant and adjuvant nivolumab or surgery alone. The use of preoperative nivolumab to help prime the immune system prior to surgical resection is an intriguing aspect to this study. Similarly, for patients with metastatic disease, the ADAPTeR study, a phase II single-arm clinical trial, has been initiated in the UK (NCT02446860). This study will assess the number of adverse events in patients in order to determine the safety of nivolumab given both before and after CN in patients with clear cell mRCC. In the purely adjuvant space, the IMmotion010 phase III randomized, placebo-controlled clinical trial is recruiting patients who previously underwent a radical or partial nephrectomy with lymphadenectomy for RCC with a high risk of metastatic progression or have undergone metastasectomy (NCT03024996). Atezolizumab will be given to the treatment group for 1 year, and the primary outcome is DFS.

## Conclusion

Surgical options for patients with advanced RCC, such as CN and/or metastasectomy, continue to offer excellent opportunities for disease control and increased survival. Surgeon participation in ongoing and developing clinical trials is also of paramount importance as we explore sequenced therapies consisting of neoadjuvant therapy, extirpative surgery, and adjuvant treatment. Careful patient selection is needed to ensure a favorable risk/benefit ratio for all patients, whether they are receiving treatment on study or off. Therefore, all patients presenting with advanced or metastatic RCC should undergo a meticulous work-up and careful staging of their disease. A thorough multidisciplinary evaluation of the patient and his or her therapeutic options is necessary to ensure that each oncology subspecialty is able to add their expertise and create an optimal and individualized treatment plan.

## Author Contributions

All authors were involved in the conception, preparation, and critical review of this manuscript.

## Conflict of Interest Statement

The authors declare that the research was conducted in the absence of any commercial or financial relationships that could be construed as a potential conflict of interest. The reviewer, JB, and the handling editor declared their shared affiliation, and the handling editor states that the process nevertheless met the standards of a fair and objective review.
